# Functional Connectivity Analysis and Detection of Mental Fatigue Induced by Different Tasks Using Functional Near-Infrared Spectroscopy

**DOI:** 10.3389/fnins.2021.771056

**Published:** 2022-03-15

**Authors:** Yaoxing Peng, Chunguang Li, Qu Chen, Yufei Zhu, Lining Sun

**Affiliations:** ^1^The Key Laboratory of Robotics System of Jiangsu Province School of Mechanical Electric Engineering Soochow University, Suzhou, China; ^2^Mathematics Teaching and Research Section, Basic Course Department, Communication Sergeant School of Army Engineering University, Chongqing, China

**Keywords:** mental fatigue detection, functional near-infrared spectroscopy, functional network characteristics, functional connectivity, common signs of fatigue tasks

## Abstract

**Objectives:**

The objective of this study was to investigate common functional near-infrared spectroscopy (fNIRS) features of mental fatigue induced by different tasks. In addition to distinguishing fatigue from non-fatigue state, the early signs of fatigue were also studied so as to give an early warning of fatigue.

**Methods:**

fNIRS data from 36 participants were used to investigate the common character of functional connectivity network corresponding to mental fatigue, which was induced by psychomotor vigilance test (PVT), cognitive work, or simulated driving. To analyze the network reorganizations quantitatively, clustering coefficient, characteristic path length, and small worldness were calculated in five sub-bands (0.6–2.0, 0.145–0.600, 0.052–0.145, 0.021–0.052, and 0.005–0.021 Hz). Moreover, we applied a random forest method to classify three fatigue states.

**Results:**

In a moderate fatigue state: the functional connectivity strength between brain regions increased overall in 0.021–0.052 Hz, and an asymmetrical pattern of connectivity (right hemisphere > left hemisphere) was presented. In 0.052–0.145 Hz, the connectivity strength decreased overall, the clustering coefficient decreased, and the characteristic path length increased significantly. In severe fatigue state: in 0.021–0.052 Hz, the brain network began to deviate from a small-world pattern. The classification accuracy of fatigue and non-fatigue was 85.4%. The classification accuracy of moderate fatigue and severe fatigue was 82.8%.

**Conclusion:**

The preliminary research demonstrates the feasibility of detecting mental fatigue induced by different tasks, by applying the functional network features of cerebral hemoglobin signal. This universal and robust method has the potential to detect early signs of mental fatigue and prevent relative human error in various working environments.

## Introduction

Long-term cognitive tasks and attention tasks may lead to mental fatigue, which is usually manifested by decreased attention, slower reaction times, and increased aversion to tasks (Boksem et al., 2006). Mental fatigue and its related decline in brain physiological function represent an important social problem, leading to reduced productivity (Tanabe and Nishihara, 2004), impaired motor and cognitive task execution (Sharma et al., 2019), reduced risk alertness (Saxby et al., 2013), and an increased incidence of accidents (Nilsson et al., 1997). To cope with these adverse but preventable consequences caused by mental fatigue, a reasonable and accurate assessment of mental fatigue degree is required. Precise assessment of fatigue degree would allow developing better strategies to arrange the work intensity and reduce potential errors or work accidents.

It is noteworthy that the signs of mental fatigue will also be different under different task situations (Ream and Richardson, 1996). Due to the complexity of the human environment, a large number of internal or external causes can lead to mental fatigue. At the same time, mental fatigue and sleepiness are often difficult to distinguish. Although involving very different concepts, they are both related in the impairment of attention, vigilance and cognitive performance (Neu et al., 2011), high degree of internal consistency among different dimensions of subjective fatigue (Matthews and Desmond, 1998), and evolution that is parallel. This paper does not want to clearly distinguish the differences between mental fatigue and sleepiness, but to explore the universal character of mental fatigue in the realistic complex environment and improve the reliability of mental fatigue indication methods.

Mental fatigue is a complex process involving the changes in multiple brain regions related to tasks, including local and global scale changes (Sun et al., 2017). Therefore, functional connectivity analysis is one of the ideal methods used to research on the mechanism of mental fatigue. A lot of prior neuroimaging studies using EEG, fMRI, or fNIRS have shown that mental fatigue is related to the deviation and reorganization of functional connectivity (Esposito et al., 2014; Qi et al., 2019; Zhang et al., 2020). In addition, graph theoretical analysis methods have been widely used in quantitative research on connectivity network structure. Sun et al. (2014a) found that in a 20-min continuous attention task, the characteristic path length of the brain network was related to the decline in task performance, and the small worldness decreased during the task. Chua et al. (2017) induced mental fatigue through a simulated driving task and found that in a 1-h task, the clustering coefficient of the brain functional network increased, and the characteristic path length decreased. In short, local clustering and inter-regional connectivity will change according to mental fatigue. These characteristics may reflect cognitive performance during fatigue.

These studies have well summarized the mechanism of functional connectivity reorganization and evolution according to mental fatigue state and effectively identify fatigue state. However, these changes can distinguish fatigue from non-fatigue state, which is not enough to give an early warning before excessive fatigue. At the same time, most studies research mental fatigue under single induced task. Considering the complexity of realistic environment, it is not applicable in guiding a universal and robust mental fatigue detection method. Therefore, it is necessary to find the common change rules of brain activity concerning the development of mental fatigue in complex work environments and use them to provide scientific methods and theoretical support for fatigue monitoring and prevention.

The main purpose of this paper is to explore the common characteristics of the functional brain network corresponding to different mental fatigue states under complex fatigue-induced conditions. Therefore, we repeated three fatigue-inducing tasks, twice in the afternoon and evening, to simulate the complex state of mental fatigue that may occur in reality, and the fatigue may include the disturbance of drowsiness. Multidimensional Fatigue Inventory (MFI-20) was used to classify three mental fatigue levels: non-fatigue, moderate fatigue, and severe fatigue. The correlation between mental fatigue and cognitive decline was measured by behavioral test results. Functional near-infrared spectroscopy is a non-invasive neuroimaging technique allowing the measurement of variations in blood oxygenation in cortical areas (Borragán et al., 2019) with acceptable spatial and tolerance to movements. fNIRS is appropriate to track changes in brain connectivity and reliably reflect cognitive load (Herff et al., 2014). This study is based on the fNIRS method to detect the blood oxygen information of the cerebral cortex throughout the experimental process, construct a functional network in five frequency bands, and quantitatively analyze the structure of a fatigue-related functional network by the network analysis method, and at the same time, extracting the characteristics for fatigue classification, so as to provide reference for cross-task mental fatigue early warning method.

## Materials and Methods

### Participants

The fatigue experiment recruited 36 undergraduate and graduate students (20 ± 2 years old, male:female = 27:9) from Soochow University. The participants were right-handed, in good health, and had no history of mental illness or cerebrovascular disease. Participants were not allowed to use medications, take a nap, and consume caffeine or stimulus drinks on the day of the experiment. All participants signed an informed consent before the experiment.

### Experiment Procedure

**FIGURE 1:**
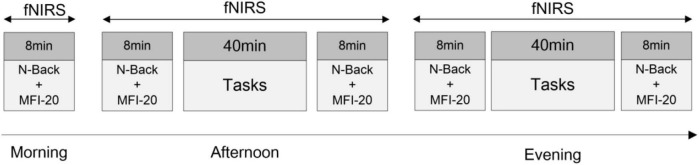
Experimental procedure. About 8 min/person in the morning. The afternoon experiment and evening experiment are about 1 h/person.

Participants were randomly divided into three groups for different fatigue-inducing experiments, including Psychomotor Vigilance Test (PVT), cognitive work, or simulated driving. Participants were asked to respect normal sleep schedules before the experiment. Each participant repeated the test three times: before starting the day’s work in the morning (7:00–8:00), in the afternoon (14:00–17:00), and before sleeping in the evening (20:00–23:00). The specific process is shown in Figure 1. In the morning, the n-back behavioral test was conducted, then the Multidimensional Fatigue Inventory (MFI-20) was filled out (8 min in total). In the afternoon and evening, the above behavioral tests and scale filling were carried out before and after the task. Cortical brain activity changes during the whole experiment were recorded using fNIRS.

### Material and Tasks

**FIGURE 2:**
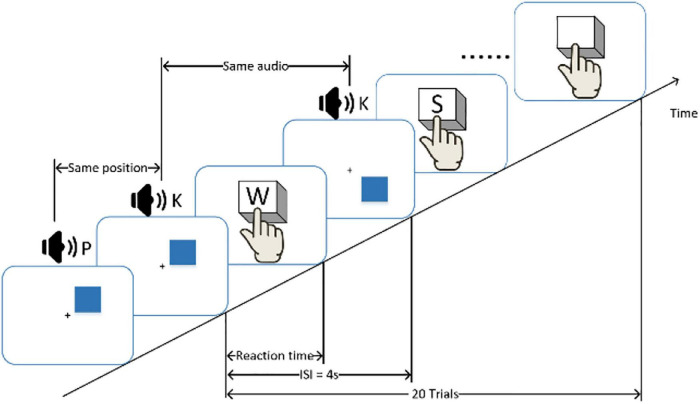
A schematic diagram of the n-back experiment. Participants were required to respond to the same consecutive stimulus and press the button as quickly as possible. The inter-stimulus interval (ISI) of the stimulus is 3 s.

Before and after the fatigue-inducing task, the participants filled in the Multidimensional Fatigue Inventory (Smets et al., 1995) and conducted the n-back (*n* = 1) behavioral test to evaluate cognition and vigilance performance associated with mental fatigue. The n-back task requires coordinated work across multiple brain regions (Cohen and D’Esposito, 2016). During the n-back test, the monitor displayed a blue square every 3S and broadcasted one random letter at the same time. Whenever a location of a square matched the location of the square presented one instance earlier, participants were asked to press the “W” key on a computer keyboard. Whenever an audio matched the last audio broadcast, participants were asked to press the “S” key on a computer keyboard. The schematic diagram of N-back is shown in Figure 2. The reaction time and accuracy of each comparison task were recorded as the basis for judging cognitive performance and vigilance.

#### Mental Fatigue Task

Mental fatigue induction tasks include psychomotor vigilance test (PVT), cognitive work, or simulated driving. PVT test is a simple reaction time test with high stimulus load. Participants were required to pay attention to the monitor. When the stimulus (red dot) was displayed in the middle of the monitor, they were supposed to respond with a button press on the keyboard as quickly as possible. In the test, inter-stimulus interval (ISI) was random and varied, ranging from 5 to 10 s. PVT task in this study was used to simulate passive mental fatigue caused by long-time simple attention tasks (Körber et al., 2015). Cognitive work included mathematical calculation tasks and foreign language reading tasks corresponding to the level of the participants, so as to simulate the mental fatigue state after the realistic learning task. A semi-immersive simulated driving task was designed. The route of the task included a motorway and a rural road. Simulated driving tasks were used to simulate passive fatigue caused by task underload under highly predictable and stable driving conditions (Larue et al., 2011; Li et al., 2016) and active fatigue caused by resource depletion due to high attention demand (Smit et al., 2004).

#### Functional Near-Infrared Spectroscopy Acquisition

**FIGURE 3:**
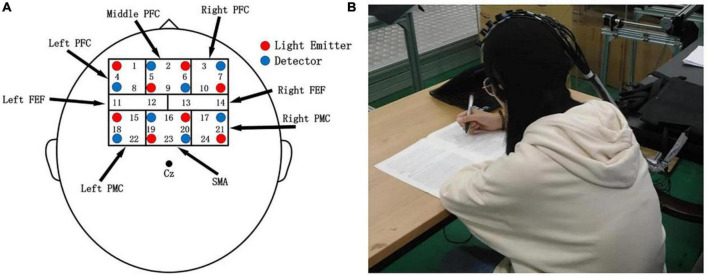
**(A)** Probe and functional brain region distribution map; red and blue numbers represent sources and detectors, respectively. **(B)** Experimental environment.

This study used a multichannel fNIRS system (NirSmart, HuiChuang, Beijing, China) to record cortical brain activity. According to Brodmann’s anatomical region system of cerebral cortex, a 4 * 4 headgear layout was designed, including 24 effective test channels composed of eight light emitters and eight light receivers, covering the prefrontal cortex (PFC), frontal eye field (FEF), supplementary motor cortex (SMA), and premotor cortex (PMC), four brain functional regions. In the experiment, the 10–20 system (Okamoto et al., 2004) was used as the positioning standard to locate the brain functional area. The channel distribution of specific brain imaging is shown in Figure 3A. The experimental environment is shown in Figure 3B. The sampling frequency was 16 Hz.

### Data Analysis

Considering the different physiological information contained in different frequency bands of blood oxygen signal (Stefanovska et al., 1999), the Chebyshev bandpass filter was used to filter the original blood oxygen signal to (I, 0.005–0.021 Hz; II, 0.021–0.052 Hz; III, 0.052–0.145 Hz; IV, 0.145–0.600 Hz, V, 0.6–2.0 Hz) five frequency bands, corresponding to the physiological information of cardiac activity, respiratory activity, myogenic activity, neurogenic activity, and endogenic activity contained in hemoglobin signal. At the same time, the influence of high-frequency noise was eliminated.

In order to evaluate the changes in activity indicators in different cerebral cortex regions, this paper divides eight regions of interest (ROI) (as shown in Table 1) and calculates the overall blood oxygen concentration of each ROI brain region, which could reduce the individual difference caused by head size. Considering the differences in individual blood oxygen active channels, the weighted average method based on entropy weight was used to calculate the blood oxygen concentration of ROI. The calculation formula is as follows:


(1) $$y=\sum_{j}X_{ij}\omega_{j}\left(i=1,2,\ldots ,N,j=1,2,\ldots ,M\right)$$

**TABLE 1:** The division of region of interest (ROI).

Name of ROI	Channels included
L-PFC	1, 4, 8
M-PFC	2, 5, 6, 9
R-PFC	3, 7, 10
L-FEF	11, 12
R-FEF	13, 14
SMA	15, 18, 22
L-PMC	16, 19, 20, 23
R-PMC	17, 21, 24

*L, M, R represent left, medial, right, respectively. For example, L-PFC represents the left part of PFC.*

*X* represents the *N* rows, *M* columns matrix composed of the original hemoglobin concentration, and ω_j_ represents the weight of each channel calculated by information entropy of channel blood oxygen concentration (Liu et al., 2017).

In order to study the dynamic interaction between regions in the process of cortical activity, Pearson correlation coefficient was used to calculate the correlation of blood oxygen concentration in each ROI during the task, and a weighted undirected network was constructed to quantitatively evaluate the changes in functional connectivity in brain-related regions corresponding to different fatigue levels. This paper calculated the clustering coefficient, characteristic path length, and small worldness. The clustering coefficient is the degree of local correlation of the network, characteristic path length is a measure of connectivity between the whole brain interval, and the small worldness reflects the unity of the overall information transmission of the network and the local high aggregation (Tononi et al., 1998; Watts and Strogatz, 1998).

In a weighted network, for node *u*, the node clustering coefficient is defined as:


(2) $$C_{u}=\frac{1}{\text{deg}\left(u\right)\left(\deg\left(u\right)-1\right)}\sum_{vw}{\left(\hat{w_{uv}}\hat{w_{uw}}\hat{w_{vw}}\right)}^{1/3}$$

where deg (*u*) is the degree of *u*. For weighted graphs, clustering coefficient is defined as the geometric average of the subgraph edge weights. The edge weights $\hat{w_{\text{uv}}}$ are normalized by the maximum weight in the network $\hat{w_{\text{uv}}}=w_{\text{uv}}/\text{max}\left(w\right)$ (Onnela et al., 2005).

The clustering coefficient of analyzed network is defined as the mean of the clustering coefficients of all nodes:


(3) $$C=\frac{1}{n}\sum_{v\in G}C_{v}$$

The path length *d*_ij_ from the node *i* to node *j* is defined as the sum of the edge lengths along this path, where the length of each edge was obtained by computing the reciprocal of the edge weight. The characteristic path length of a weight directed graph was defined as the smallest sum of the edge lengths throughout all the possible paths (Sun et al., 2014a):


(4) $$L=\frac{1}{n\left(n-1\right)}\sum_{i\neq j}d_{ij}$$

Small worldness:


(5) $$\sigma =\frac{C_{real}}{C_{random}}/\frac{L_{real}}{L_{random}}$$

The small worldness could be summarized from the normalized clustering coefficient (γ = *C*_real_/*C*_random_) and the normalized characteristic path length (λ = *L*_real_/*L*_random_), where *C*_real_ and *L*_real_ are the clustering coefficient and characteristic path length of the analyzed network, *C*_random_ and *L*_random_ are the mean clustering coefficient and the mean characteristic path length of 100 matched random networks, respectively.

To compare the topological structure of functional connectivity without bias from different average connectivity difference (Sun et al., 2014a), the small worldness of unweighted network was calculated. Sparsity is defined as the ratio of the number of actual edges to the number of all possible edges in a fully connected network. A sparsity of 0.45 was adopted to convert the full connection matrix to a sparse network.

Three kinds of blood oxygen information, including oxyhemoglobin, deoxyhemoglobin, and total oxyhemoglobin, were recorded in the experiment. Characteristics of brain functional network were calculated under the three kinds of blood oxygen in this study.

### Statistics

All statistical analyses were performed using SPSS version 25.0 (IBM Corp., Armonk, NY, United States). For MFI-20 scores, N-back test scores, functional connectivity strength, and network metrics, the primary result was analyzed by two-way analysis of variance (ANOVA) with three fatigue levels [L1 (non-fatigue), L2 (moderate fatigue), and L3 (severe fatigue)], and the three tasks (PVT, cognitive work, and simulated driving) as factors. If the significant main effects were on the fatigue level, but no interactions were detected, Tukey-Kramer’s *post-hoc* test was used to locate differences between fatigue levels. For all analyses, the statistical significance was set at *p* < 0.05.

### Mental Fatigue Classification

The levels of mental fatigue were defined by the score of MFI-20 scale and behavioral test. A clustering analysis of unsupervised k-means was performed on MFI-20 scale score, and the participants were grouped into non-fatigue and fatigue. According to the results of clustering analysis, taking the scale score of 2.57 as the threshold, the participants with a scale score less than 2.57 were classified as non-fatigue, and the participants with a score more than or equal to 2.57 were classified as fatigue. Then according to the score of n-back behavioral task (reaction time/accuracy) and MFI-20, the fatigue participants were further grouped into moderate fatigue and severe fatigue. This study took the scale score threshold from 2.57 to 5.00 with step of 0.01; the behavioral test scores of the grouped participants were statistically analyzed. When the threshold was 3.00, the statistical difference of behavioral test scores between moderate fatigue and severe fatigue participants was the largest, so the participants with a score more than or equal to 3.00 were divided into severe fatigue.

### Machine Learning Method

To verify the feasibility of mental fatigue detection based on fNIRS data, we performed classification of mental fatigue states based on the functional connectivity strength and characteristics of brain functional network. This paper classified three mental fatigue levels: non-fatigue, moderate fatigue, and severe fatigue. The features include Pearson correlation coefficient (r) between eight ROI(3 hemoglobins * 5 frequency bands * 28 channel pairs), characteristic path length and clustering coefficient of brain functional network(3 hemoglobins * 5 frequency bands * 2 network characteristics), and 15 time-domain features(3 hemoglobins * 5 frequency bands * 8 ROI * 15 time-domain feature). The time-domain features include mean, standard deviation, coefficient of variation, energy, range, skewness, kurtosis and peak, Hjorth parameter, information entropy, root mean square, kurtosis factor, waveform factor, pulse factor, and margin factor. Considering the unbalanced number of three-level participants and high feature dimension, this study used the random forest algorithm based on Python library sklearn and random forest classifier. Because of the large number of available features, the key features were selected based on the Gini coefficient, and then the features were further screened, and the parameters of random forest classifier were optimized by genetic algorithm.

## Results

### Mental Fatigue Degree and Behavioral Task Performance

**FIGURE 4:**
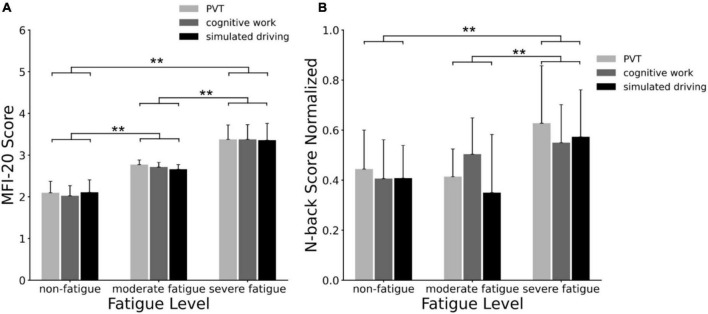
**(A)** Multidimensional Fatigue Inventory (MFI-20) scale scores correspond to non-fatigue, moderate fatigue, and severe fatigue grade participants; the mean and standard deviation of each group were calculated; **(B)** behavioral task performance corresponded to non-fatigue, moderate fatigue, and severe fatigue participants. The behavioral task scores (reaction time/accuracy) were normalized, and the mean value and standard deviation of each group were calculated. **p < 0.01.

The MFI-20 scale scores corresponding to participants of three fatigue levels are shown in Figure 4A. The ANOVA results revealed a significant main effect of fatigue level (*F* = 305.584; *p* < 0.001; η^2^ = 0.795) on MFI-20 scores, but no significant main effect of task (*p* = 0.715) or interaction between fatigue level and task (*p* = 0.844) was found. *Post-hoc* analyses indicated significant increases in MFI-20 score between non-fatigue and moderate fatigue (*p* < 0.001), between moderate fatigue and severe fatigue (*p* < 0.001), and between non-fatigue and severe fatigue (*p* < 0.001).

The N-back task scores corresponding to participants of three fatigue levels are shown in Figure 4B. The ANOVA results revealed a significant main effect of fatigue level (*F* = 15.759; *p* < 0.001; η^2^ = 0.166) on N-back scores, and no main effect of task (*p* = 0.286) or interaction between fatigue level and task (*p* = 0.259) was significant. *Post-hoc* analyses indicated significant increases on N-back score between non-fatigue and severe fatigue (*p* < 0.001), and between moderate fatigue and severe fatigue (*p* < 0.001).

### Functional Connectivity Network

**FIGURE 5:**
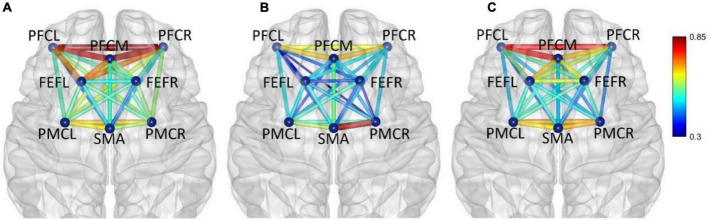
Frequency band I (0.005–0.021 Hz), **(A)** non-fatigue, **(B)** moderate fatigue, and **(C)** severe fatigue. The average cortical functional connectivity network contains eight ROI brain regions. The width and color of the line represent the strength of the connectivity. Functional connectivity was visualized with the BrainNet Viewer (http://www.nitrc.org/project/bnv/).

**FIGURE 6:**
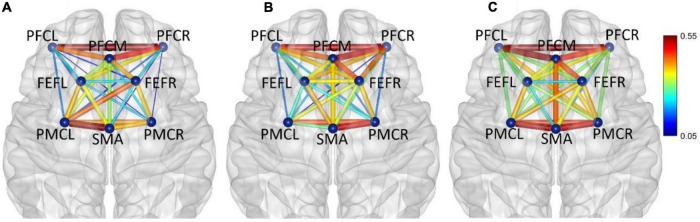
In frequency band II (0.021–0.052 Hz), non-fatigue **(A,B)** moderate fatigue, and **(C)** severe fatigue. The average cortical functional connectivity network contains eight ROI brain regions. The width and color of the line represent the strength of the connectivity.

**FIGURE 7:**
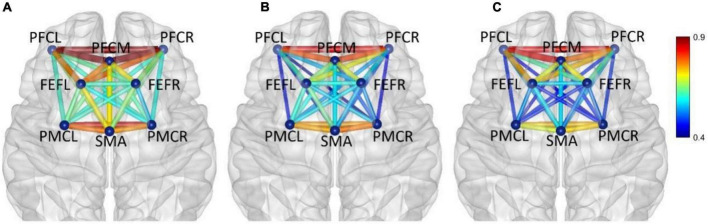
In frequency band III (0.052–0.145 Hz), non-fatigue **(A,B)** moderate fatigue, **(C)** severe fatigue. The average cortical functional connectivity network contains 8 ROI brain regions. The width and color of the line represent the strength of the connectivity.

**FIGURE 8:**
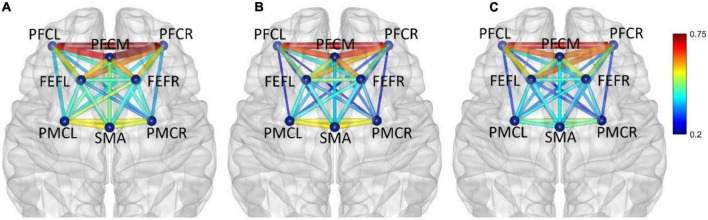
In frequency band IV (0.145–0.600 Hz), non-fatigue **(A,B)** moderate fatigue, **(C)** severe fatigue. The average cortical functional connectivity network contains eight ROI brain regions. The width and color of the line represent the strength of the connectivity.

**FIGURE 9:**
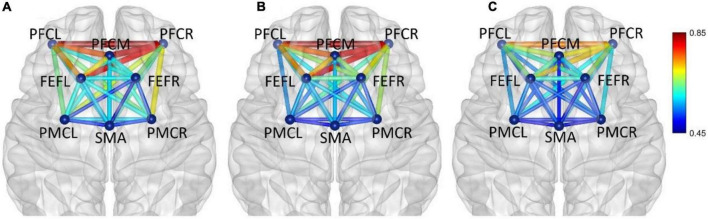
In frequency band V (0.6–2.0 Hz), non-fatigue **(A,B)** moderate fatigue, and **(C)** severe fatigue. The average cortical functional connectivity network contains eight ROI brain regions. The width and color of the line represent the strength of the connectivity.

In five frequency bands, the average functional connectivity network corresponding to the three mental fatigue levels are shown in Figures 5–9, respectively. In each band, two-way ANOVA was conducted on connectivity strength with the two factors: fatigue level (non-fatigue, moderate fatigue, and severe fatigue) and task (PVT, cognitive work, and simulated driving). When there was a significant main effect on fatigue level on one connectivity, there was no significant interaction between fatigue level and task; the ANOVA results of that connectivity will be shown below.

In frequency band I (0.005–0.021 Hz), the average functional connectivity network corresponding to the three mental fatigue levels are shown in Figure 5. The results of ANOVA revealed a significant main effect of fatigue level on the correlations of PFCM_PFCR (*F* = 4.655, *p* = 0.010, η^2^ = 0.026) and FEFR_PMCR (*F* = 4.276, *p* = 0.015, η^2^ = 0.024), and no significant interaction between fatigue level and task (*p* > 0.311) was found. *Post-hoc* analyses indicated a significant decrease in FEFR_PMCR connection between non-fatigue and moderate fatigue (*p* = 0.029), and a significant decrease in PFCM_PFCR connection between non-fatigue and severe fatigue (*p* = 0.030). From non-fatigue to moderate fatigue, the network connectivity decreased overall, especially between the regions of PFC and FEF, and between the regions of PFC and PMC. However, the connectivity strength remained relatively constant among PFCL, PFCM, and PFCR, as well as between the regions of SMA and PMC. From moderate fatigue to severe fatigue, the network connectivity increased overall, and a relatively compact connectivity was maintained between the left PFC and other brain regions, especially between PFC and FEF.

In frequency band II (0.021–0.052 Hz), the average functional connectivity network corresponding to the three mental fatigue levels are shown in Figure 6. The results of ANOVA revealed a significant main effect of fatigue level on the correlations of PFCL_FEFR (*F* = 5.994, *p* = 0.003, η^2^ = 0.034) and PFCR_PMCR (*F* = 3.639, *p* = 0.027, η^2^ = 0.021); no significant interaction between fatigue level and task (*p* > 0.095) was found. *Post-hoc* analyses indicated a significant increase in PFCL_FEFR connection between non-fatigue and moderate fatigue (*p* = 0.013), and between non-fatigue and severe fatigue (*p* = 0.049), and a significant increase in PFCR_PMCR connection between non-fatigue and severe fatigue (*p* = 0.029). From non-fatigue to moderate fatigue, the overall connectivity strength of the right hemisphere increased. The average functional connectivity strength of the left and right hemispheres was calculated. The connectivity strength of the right hemisphere is greater than that of the left hemisphere (*p* = 0.005). When severe fatigue occurred, there was no significant difference in the connectivity strength between the left and right hemispheres (*p* = 0.273).

In frequency band III (0.052–0.145 Hz), the average functional connectivity network corresponding to the three mental fatigue levels are shown in Figure 7. The results of ANOVA revealed the significant main effect of fatigue level on the correlations of PFCL_PFCM, PFCL_PFCR, PFCL_FEFL, PFCL_PMCL, PFCL _PMCR, PFCM_PFCR, PFCM_PMCL, PFCR_SMA, PFCR_PM CL, PFCR_PMCR, FEFL_SMA, FEFL_PMCL, FEFL_PMCR, FEFR_PMCL, and PMCL_PMCR; no significant interaction between fatigue level and task (*p* > 0.051) was found. The details of ANOVA are shown in Table 2.

**TABLE 2:** Functional connectivity with significant main effect of fatigue level in band frequency III.

Functional connectivity	*F*	*p*-value	Partial eta squared (η^2^)
PFCL_PFCM	5.835	0.003	0.033
PFCL_PFCR	5.013	0.007	0.028
PFCL_FEFL	10.848	<0.001	0.059
PFCL_PMCL	6.678	0.001	0.037
PFCL_PMCR	10.571	<0.001	0.058
PFCM_PFCR	10.452	<0.001	0.057
PFCM_PMCL	12.306	<0.001	0.067
PFCR_SMA	4.975	0.007	0.028
PFCR_PMCL	4.610	0.011	0.026
PFCR_PMCR	4.977	0.007	0.028
FEFL_SMA	7.640	0.001	0.042
FEFL_PMCL	4.068	0.018	0.023
FEFL_PMCR	3.901	0.021	0.022

*No interaction was significant (all p > 0.05) on functional connectivity above.*

*Post-hoc* analyses indicated significant decreases in PFCL_PFCM, PFCL_FEFL, PFCL_PMCL, PFCL_PMCR, PFCM _PFCR, PFCR_SMA, PFCR_PMCR, and PMCL_PMCR connections between non-fatigue and moderate fatigue, decreases in PFCL_PFCR, PFCL_PMCR, PFCM_PFCR, PFCM _PMCL, PFCR_PMCL, FEFL_SMA, FEFL_PMCL, FEFL_PMCR, FEFR_PMCL connections between non-fatigue and severe fatigue, increase in PFCL_FEFL connection between moderate fatigue and severe fatigue, and a decrease in PFCM_PMCL, PMCL_PMCR connection between moderate fatigue and severe fatigue. The details of *post-hoc* analyses are shown in Table 3. From non-fatigue to moderate fatigue, the connectivity of the whole brain network decreased significantly. From moderate fatigue to severe fatigue, the whole network only maintained relatively strong connectivity between the regions of PFC and FEF.

**TABLE 3:** *Post-hoc* analyses among fatigue levels in band frequency III.

Fatigue level	Fatigue level	Functional connectivity	Mean difference (I–J)	*p*-values
L1	L2	PFCL_PFCM	0.097	0.002
		PFCL_FEFL	0.168	<0.001
		PFCL_PMCL	0.137	0.016
		PFCL_PMCR	0.202	<0.001
		PFCM_PFCR	0.099	0.007
		PFCR_SMA	0.149	0.010
		PFCR_PMCR	0.137	0.021
		PMCL_PMCR	0.100	0.007
	L3	PFCL_PFCR	0.061	0.016
		PFCL_PMCR	0.157	0.002
		PFCM_PFCR	0.121	<0.001
		PFCM_PMCL	0.203	<0.001
		PFCR_PMCL	0.126	0.015
		FEFL_SMA	0.147	<0.001
		FEFL_PMCL	0.116	0.027
		FEFL_PMCR	0.119	0.019
		FEFR_PMCL	0.144	0.013
L2	L3	PFCL_FEFL	−0.141	0.001
		PFCM_PMCL	0.156	0.009
		PMCL_PMCR	0.116	<0.001

In frequency band IV (0.145–0.600 Hz), the average functional connectivity network corresponding to the three mental fatigue levels are shown in Figure 8. The results of ANOVA revealed a significant main effect of fatigue level on the correlation of PFCM_PMCL (*F* = 4.945, *p* = 0.008, η^2^ = 0.028); no significant interaction between fatigue level and task (*p* = 0.165) was found. *Post-hoc* analyses indicated a significant decrease in PFCM_PMCL connection between non-fatigue and severe fatigue (*p* = 0.034). From non-fatigue to moderate fatigue, the functional connectivity of the whole brain network was weakened. From moderate fatigue to severe fatigue, the whole network only maintained relatively strong connectivity between the regions of PFC and FEF.

In frequency band V (0.6–2.0 Hz), the average functional connectivity network corresponding to the three mental fatigue levels are shown in Figure 9. The functional connectivity of the whole brain network did not change significantly. From moderate fatigue to severe fatigue, the functional connectivity of the whole brain network decreased, especially between PFC and other regions of the brain.

### Characteristics of Brain Functional Network

**FIGURE 10:**
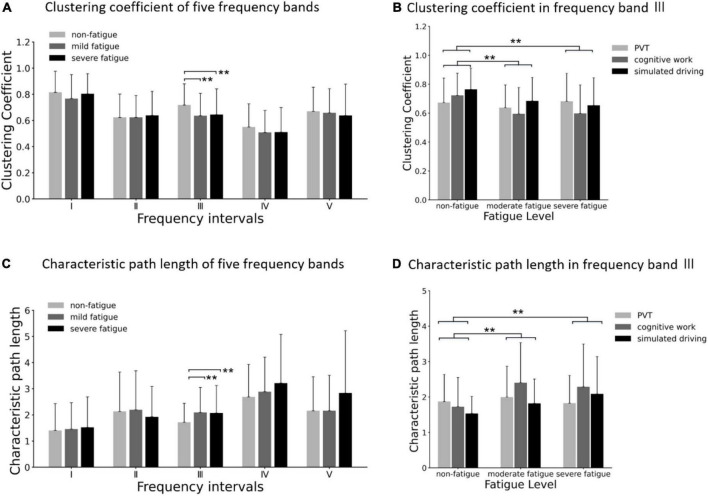
**(A)** Comparison of clustering coefficient of three fatigue levels in five frequency bands. **(B)** Results of *post-hoc* analyses of clustering coefficient in frequency band III (0.052–0.14 5 Hz). **(C)** Comparison of characteristic path length of three fatigue levels in five frequency bands. **(D)** Results of *post-hoc* analyses of characteristic path length in frequency band III (0.052–0.145 Hz). Vertical bars are standard errors scores. **p < 0.01.

Comparison of clustering coefficient among three fatigue levels in five frequency bands are shown in Figure 10A. In frequency band III, the significant main effects of fatigue level on clustering coefficient (*F* = 8.670; *p* < 0.001; η^2^ = 0.048) are presented, but no significant interaction between fatigue level and task (*p* > 0.05) was found, as shown in Figure 10B. *Post-hoc* analyses confirmed the decrease in clustering coefficient between non-fatigue and moderate fatigue (*p* = 0.002), and between non-fatigue and severe fatigue (*p* = 0.002).

Comparison of characteristic path length among three fatigue levels in five frequency bands is shown in Figure 10C. In frequency band III, the significant main effects of fatigue level on characteristic path length (*F* = 8.670; *p* < 0.001; η^2^ = 0.048) are presented, but no significant interaction between fatigue level and task (*p* > 0.05) was found, as shown in Figure 10D. *Post-hoc* analyses confirmed the increase in characteristic path length between non-fatigue and moderate fatigue (*p* = 0.006), and between non-fatigue and severe fatigue (*p* = 0.003).

**FIGURE 11:**
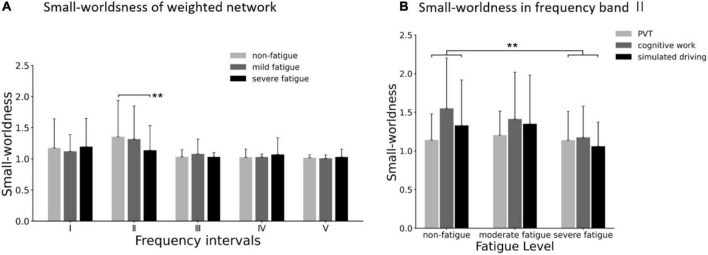
**(A)** Comparison of small worldness in weighted network of three fatigue levels in five frequency bands. **(B)** Results of *post-hoc* analyses of small worldness in weighted network in frequency band II (0.021–0.052 Hz). Vertical bars are standard errors scores. **p < 0.01.

As for each fatigue level, average small worldness of weighted networks was larger than 1 in all the five frequency bands (as shown in Figure 11A), which indicated that all the connectivity networks in the three fatigue levels displayed small-world characteristics. In frequency band II, the significant main effect of fatigue level on small worldness was presented (*F* = 4.290; *p* = 0.015; η^2^ = 0.028), but no significant main effect of task (*p* = 0.059) or interaction between fatigue level and task (*p* = 0.120) was found. As shown in Figure 11B. *Post-hoc* analyses confirmed the decrease in small worldness between non-fatigue and severe fatigue (*p* = 0.042).

**FIGURE 12:**
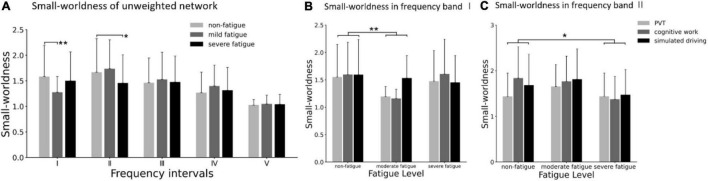
**(A)** Comparison of small worldness in unweighted network of three fatigue levels in five frequency bands. **(B)** Results of *post-hoc* analyses of small worldness in unweighted network in frequency band I (0.005–0.021 Hz). **(C)** Results of *post-hoc* analyses of small worldness in unweighted network in frequency band II (0.021–0.052 Hz). Vertical bars are standard errors scores. *p < 0.05; **p < 0.01.

Considering the possible influence of the difference average connectivity strength among the three fatigue levels on the network topological structure, a common sparsity has been considered in each weighted network through a dynamic threshold. In other words, all unweighted networks were guaranteed to have the same number of edges. The small worldness of unweighted networks are shown in Figure 12B. In frequency bands I and II, the significant main effect of fatigue level on unweighted small worldness was presented (band I: *F* = 3.812; *p* = 0.023; η^2^ = 0.030; band II: *F* = 4.064; *p* = 0.018; η^2^= 0.032), but no significant main effect of task (*p* > 0.419) or interaction between fatigue level and task (*p* > 0.101) were found. In frequency band I, *post-hoc* analyses confirmed the decrease in small worldness between non-fatigue and moderate fatigue (*p* = 0.037). In frequency band II, *post-hoc* analyses confirmed the decrease in unweighted small worldness between non-fatigue and severe fatigue (*p* = 0.010).

### Mental Fatigue Classification

In this study, the functional connectivity strength, characteristics of brain functional network, and time-domain characteristics of blood oxygen signal were used as features to classify the mental fatigue levels. The classification accuracy of non-fatigue and fatigue was 85.4%, the recall of fatigue was 89.3%, and the F1 score was 87.5% with a fivefold cross-validation. The classification accuracy of moderate fatigue and severe fatigue was 82.8%, the recall of severe fatigue was 90.5%, and the F1 score is 85.7% with a fivefold cross-validation.

## Discussion

In this study, there were three fatigue-inducing tasks: psychomotor vigilance test (PVT), cognitive work, and simulated driving. Based on fNIRS, cerebral hemoglobin information under different fatigue levels was recorded to construct the functional networks. Changes in functional connectivity and functional network reorganization under different fatigue levels were studied by graph theoretical analysis methods. The main findings are as follows:

As for moderate fatigue, the adequate behavioral task performances of participants were maintained, and there was no significant worsening in response time and accuracy. In frequency band II associated with neurogenic activity, the functional connectivity of the right hemisphere was generally enhanced; an asymmetrical pattern of connectivity (right hemisphere > left hemisphere) was presented. The most significant reorganization of functional connectivity network was in frequency band III associated with myogenic activity, the functional connectivity strength decreased overall, the clustering coefficient of brain network decreased, and the characteristic path length increased significantly.

As for severe fatigue, behavioral test performance decreased significantly. In frequency band II associated with neurogenic activity, the functional connectivity strength increased overall, and there was no significant difference in the connectivity strength between the left and right hemispheres. Small worldness showed significant differences in frequency band II, and as fatigue deepened, the brain network structure began to deviate from the small-world pattern. In frequency band II associated with neurogenic activity, the small worldness decreased continuously, and the small worldness decreased significantly when severe fatigue occurred.

### Moderate Mental Fatigue, Task Performance, and Changes in Functional Connectivity

Functional connectivity of the brain network based on regional cerebral blood flow (rCBF) plays an important role in information transmission across regions (Liang et al., 2013). There is a tight coupling between blood supply and brain functional topology. The functional connectivity strength showed a striking spatial correlation with rCBF (Liang et al., 2013). The oscillations in frequency bands I, II, and III reflect the influence of endothelial-related metabolic activity, intrinsic neuronal activity, and myogenic activity of the vascular smooth muscle (Shiogai et al., 2010; Li et al., 2012).

Compared with the non-fatigue state, the performance of the participants in the n-back test remained adequate in moderate fatigue, and there was no significant change in response to time and accuracy. In frequency band II associated with neurogenic activity, the strength of functional connectivity in the right hemisphere increased significantly, while the strength of connectivity between the left PMC and other brain regions decreased. There was an asymmetric network pattern between the left and right hemispheres in cortical connectivity. In previous studies, in the middle of the sleep deprivation experiment, the cortical activity of the right hemisphere increased mainly, while the bilateral activity increased in the later stage of the experiment (Borragán et al., 2019), and an increase in the frontal cortex oxygenation at the start of the driving task was found (Li et al., 2009); Asymmetric brain activation according to different types of mental fatigue was also reported (Shigihara et al., 2013). Other prior studies reported a major involvement of the right hemisphere during sustained attention tasks when tasks remained relatively easy or the participants remained alert. When the cognitive load increased, unilateral activation during the task was replaced by a bilateral activation (Klingberg et al., 1997; Helton et al., 2010), and the brain can obtain additional processing ability by activating both hemispheres (Scalf et al., 2009). Our results are similar to those studies. When the fatigue degree was mild, the same as the task load, the connectivity strength of the right hemisphere increased significantly; the connectivity pattern of the left and right hemispheres presented significant differences. The connectivity pattern of the left and right hemispheres tends to be the same in severe fatigue state.

In frequency band I, the functional connectivity strength decreased overall, and the clustering coefficient decreased significantly. That means that the connectivity strength between neighbor nodes decreased, and the degree of coordination in local brain regions decreased. The decreased clustering coefficient reflected the lower processing rate of local information. The oscillations in this frequency band reflect the myogenic activity of the vascular smooth muscle, and the substances related to metabolism have a direct effect on the state of contraction of the vascular musculature (Shiogai et al., 2010). In previous studies, higher fatigue would result in more endogenic regulation, and participants with regular exercise training showed less regulation related to endothelial cell metabolic activity in fatigue tasks (Lu et al., 2019). Similar to our study, the lower clustering coefficient may be the reflection of the lower demand for endogenous regulation in moderate fatigue.

The oscillations in frequency band III (0.052–0.145 Hz) reflect the myogenic activity of cerebrovascular smooth muscle, which plays an important role in the autoregulation of cerebral blood flow (Osol, 1995; Rowley et al., 2007). In the case of moderate fatigue, the overall functional connectivity strength of this frequency band decreased, and the corresponding clustering coefficient decreased significantly. Similar to our conclusion, previous studies have shown that the functional connectivity of myogenic frequency band decreases significantly in fatigue driving (Xu et al., 2017). It was observed in the early stage of the grip strength task that the brain directed phase transfer entropy, and the number of directional connectivity of the participants undergoing regular exercise were limited, and the number of directional connectivity increased significantly when the fatigue increased in the later stage of the task (Osol, 1995). Similar to the endogenic frequency band, the availability of local resources may be the reason for the decreased connectivity strength between regions. At the same time, when the degree of fatigue deepened, the relevant areas must raise more resources from other areas to maintain task performance, which was also consistent with the increase in connectivity strength among PFC, FEF, and SMA observed in severe fatigue.

### Severe Mental Fatigue, Task Performance, and Changes in Functional Connectivity

Compared with moderate fatigue, the behavior test performance of participants decreased significantly in severe fatigue. In the endogenic frequency band I and neurogenic frequency band II, the overall functional connectivity strength was maintained or increased, which reflected the additional activation of brain interval connection in response to resource depletion (Sun et al., 2014b; Li et al., 2019). The brain required more functional connectivity to complete the same tasks, which further exacerbates mental fatigue. At the same time, it was observed that the small worldness of the weighted network decreased significantly in neurogenic frequency band II. Considering the possible influence of the difference in average functional connectivity strength among three fatigue levels on the network structure characteristics, we maintain that each network has the same sparsity through a dynamic threshold. The small worldness calculated by this method showed a similar result (as shown in Figure 12). The small worldness reflects the balance of brain network in regional local cooperation and information transmission between regions and represents the best form of brain functional network structure. The decline in performance in fatigue state is related to the deviation of the brain network from small worldness (Sun et al., 2014a; Li et al., 2019). In previous pathological studies, the loss of small-world pattern has also been observed in the patient population, such as Alzheimer’s disease (Stam et al., 2007), schizophrenia (Micheloyannis et al., 2006), etc. Small worldness is one of the key characteristics of health network. In severe fatigue, the decrease in small worldness reflected the decline in local specialized processing ability of brain network and information transmission efficiency between regions.

In frequency band IV, the overall connectivity strength decreased, only relatively weak connectivity remained between the regions of PFC and PMC, SMA. At the same time, the performance of the behavioral test decreased significantly. Anatomically, the prefrontal cortex is located at the top of the sensory and motor levels (Curtis and D’Esposito, 2003). The PFC can guide and regulate the functional connectivity pattern of brain regions by guiding attention, integrating sensory stimulation and motor planning, exert top–down influence on perceptual and sensorimotor areas (Hopfinger et al., 2000; Sarter et al., 2001). Sustained attention is a direct consequence of top-down signaling (Sun et al., 2014a). The decrease in connectivity strength between PFC, FEF, and PMC, SMA reflected that the depletion of brain resources led to the impairment of interregional information transmission efficiency, which reduced the ability of interregional cooperative problem solving and the accuracy of behavioral performance. Therefore, the behavioral task performance in severe fatigue declined.

Consistent with the decline in connectivity between regions, in terms of brain functional network characteristics, the characteristic path length in frequency band IV and frequency band V increased during severe fatigue. The change in clustering coefficient of the brain network is related to cognitive performance (Struck et al., 2021). In the state of mental fatigue, more isolation and less aggregation were observed (Li et al., 2016). Similar to our results, Sun et al. (2014a) found that the increase in the characteristic path length was related to the increase in reaction time caused by continuous attention tasks. Chen et al. (2019) induced mental fatigue through driving tasks. Significant differences in functional connectivity were observed between alert state and fatigue state. The frontal-to-parietal functional connectivity was weakened. Meanwhile, lower clustering coefficient values and higher characteristic path length values were observed in fatigue state in comparison with alert state. The characteristic path length is the embodiment of the overall connectivity of the brain network, which means that the information transmission efficiency of the cortical brain region is reduced (Watts and Strogatz, 1998). In high-frequency bands, functional connectivity mode was characterized by the decline in the overall functional connectivity strength and the limited connectivity between regions.

There are also some limitations in this study. The MFI-20 scale was used as the basis for defining the fatigue level, but there were deviations in the understanding of different people when filling in the scale. At the same time, the sensitivity of the scale to specific fatigue responses caused by different participants may be different (Di Stasi et al., 2012), which may be partially deviated from the real fatigue state of the participants. In future research, we can use a combination of a variety of subjective fatigue scales (Sun et al., 2017) or fatigue state judgment methods based on physiological signals and behavioral data.

## Conclusion

This paper investigated the common character of functional connectivity network corresponding to mental fatigue induced by different tasks and classified the fatigue levels based on the common features. With the deepening of mental fatigue, the deviation and reorganization of functional connectivity were observed, and those changes reflected the unique forms of brain network functional connectivity under different fatigue levels.

In moderate fatigue, the overall functional connectivity of the neurogenic frequency band increased significantly, and the connectivity strength of the right hemisphere was greater than that of the left hemisphere. The connectivity strength of the endogenic frequency band and the myogenic frequency band decreased, and the clustering coefficient decreased significantly; In severe fatigue, the overall functional connectivity strength of neurogenic frequency band increased, and the small worldness decreased. In the high-frequency band, only the PFC and FEF maintained close connectivity, and the characteristic path length increased.

Based on the common characteristics, the random forest classifier was used to distinguish the fatigue level induced by different tasks. The classification accuracy of non-fatigue and fatigue is 85.4%, and the recall of fatigue is 89.3%. The classification accuracy of moderate fatigue and severe fatigue was 82.8%, and the recall of severe fatigue was 90.5%. The common character of functional connectivity and preliminary fatigue discrimination results under each fatigue state prove that the findings of this study have potential application value for mental fatigue monitoring and early warning under complex conditions.

## Data Availability Statement

The raw data supporting the conclusions of this article will be made available by the authors, without undue reservation.

## Ethics Statement

The studies involving human participants were reviewed and approved by the Ethics Committee of Soochow University (ECSU) approval No. SUDA20210909H01. The patients/participants provided their written informed consent to participate in this study.

## Author Contributions

YP: acquisition of data, analysis and interpretation of data, drafting the manuscript. CL: conception and design of study, analysis, and interpretation of data, review and editing. QC, YZ, and LS: review and editing. All authors contributed to the article and approved the submitted version.

## Conflict of Interest

The authors declare that the research was conducted in the absence of any commercial or financial relationships that could be construed as a potential conflict of interest.

## Publisher’s Note

All claims expressed in this article are solely those of the authors and do not necessarily represent those of their affiliated organizations, or those of the publisher, the editors and the reviewers. Any product that may be evaluated in this article, or claim that may be made by its manufacturer, is not guaranteed or endorsed by the publisher.
